# Blood Volume and Haemoglobin Mass in Relation to Fat-Free Mass and Aerobic Capacity in Elite Junior Rowers

**DOI:** 10.3390/sports14050192

**Published:** 2026-05-07

**Authors:** Viktorija Maconytė, Loreta Stasiulė, Arvydas Stasiulis

**Affiliations:** Department of Health Promotion and Rehabilitation, Lithuanian Sports University, 44221 Kaunas, Lithuania; loreta.stasiule@lsu.lt (L.S.); arvydas.stasiulis@lsu.lt (A.S.)

**Keywords:** rowers, aerobic capacity, blood volume, haemoglobin mass, fat-free mass

## Abstract

Background: Blood volume (BV), haemoglobin mass (Hb-mass) are key determinants of blood oxygen transport. The aim of this study was to assess BV and Hb-mass in elite junior rowers and evaluate their relationship with fat-free mass (FFM) and aerobic capacity. Methods: Twenty-five males (18.4 ± 2.4 y, 1.92 ± 0.5 m, 89.3 ± 4.7 kg) and fourteen females (17.0 ± 1.9 y, 1.77 ± 0.7 m, 74.2 ± 11.3 kg) participated. BV, plasma volume (PV), and Hb-mass were assessed via CO rebreathing. Pulmonary gas exchange was measured during a graded rowing test. Results: Males had higher absolute BV (7270 ± 717 vs. 5388 ± 471 mL) and Hb-mass (1083 ± 91 vs. 720 ± 49 g). After adjusting for FFM, most differences disappeared, except PV, which remained higher in females (57.2 ± 4.8 vs. 49.9 ± 6.5 mL·FFM·kg^−1^). V̇O_2_max was higher in males in absolute (6.28 ± 0.40 vs. 4.48 ± 0.29 L·min^−1^) and body-mass-relative terms (70.2 ± 5.6 vs. 61.3 ± 7.3 mL·kg^−1^·min^−1^), but not when expressed per FFM (79.4 ± 5.3 vs. 81.1 ± 7.3 mL·FFM·kg^−1^·min^−1^). BV, PV, and Hb-mass correlated positively with V̇O_2_max in both sexes, with stronger associations in females. Conclusions: Sex differences in blood parameters among junior rowers are largely explained by FFM, except for PV, which is relatively higher in females. Stronger associations between blood variables and aerobic capacity in females suggest greater reliance on central oxygen transport.

## 1. Introduction

Olympic rowing performance is determined by aerobic and anaerobic capacity, as rowers must maintain endurance for 5.5–7 min at a distance of 2000 m [1]. Aerobic metabolism provides significant energy (from 75% to 80%) needed for rowing competition compared with anaerobic metabolism, which provides only between 12% and 30% of energy [2]. Therefore, higher V̇O_2_max is essential for better endurance and overall performance in rowing [3]. Adult world-class male rowers have high absolute V̇O_2_max values of 7 L/min [4], whereas elite junior, sub-elite senior, and elite senior rowers tend to have lower absolute V̇O_2_max values of 5–6 L/min [5]. Female rowers tend to have ~30% lower absolute V̇O_2_max values, independent of performance level [6,7,8].

High-level adult male rowers tend to have high total blood volume (BV) and total haemoglobin mass (Hb-mass) values of up to ~10 L and ~1.4 kg, respectively [9,10,11,12], whereas younger male adult rowers (aged 22–23 years) with less training experience have lower BV values of up to ~ 6.5 L [13]. Elite female rowers are ~5 L and ~0.7 kg, respectively [13]. To our knowledge, no research has explored the BV and Hb-mass in junior rowers of both sexes.

Since higher fat-free mass (FFM) and lower fat mass (FM) are linked with better rowing performance, body mass (BM) and composition should also be considered when assessing BV, Hb-mass, and V̇O_2_max. The higher absolute V̇O_2_max, BV, and Hb-mass values in rowers may be due to their higher BM and FFM than those of other athletes [14]. In addition, these physiological characteristics are influenced by training experience, performance level, and competitive background. Reference values for V̇O_2_max differ across junior, U23, and senior elite rowers, and longitudinal data in adolescent rowers show further increases in aerobic and anaerobic power with continued training and maturation. Similarly, hematological adaptations are related to endurance training status, and changes in training load may influence plasma volume, blood volume, and exercise capacity. Therefore, training age and competitive level should be considered when interpreting physiological profiles in junior rowers [5,15]. There are no data concerning elite junior female rowers.

V̇O_2_max is influenced by hematological parameters such as BV and Hb-mass, which are crucial for maintaining adequate blood flow and oxygen delivery during intense exercise [10,16]. Studies have shown that Hb-mass is related to power output, ventilatory thresholds, and V̇O_2_max, demonstrating the direct impact of haematological parameters on aerobic capacity in adult or senior rowers [10,11,17]. A study on Olympic rowers revealed that reductions in PV and BV correlated with decreased maximal power output during a graded exercise test, indicating that maintaining or increasing BV is beneficial for sustaining high aerobic capacity [10]. However, a stronger relationship between relative Hb-mass and relative V̇O_2_max was observed in trained female rowers than in trained male rowers [18]. BV has been shown to have a positive effect on V̇O_2_max after the onset of puberty [19]. However, owing to limited data, the contributions of BV and Hb-mass to aerobic capacity in male and female junior rowers remain unclear. Understanding the physiology of young rowers is crucial for optimizing their training and performance. Therefore, the aim of this study was to assess BV and Hb-mass in elite junior rowers and evaluate their relationship with fat-free mass (FFM) and aerobic capacity.

## 2. Materials and Methods

### 2.1. Participants

Thirty-nine competitive academic rowers (twenty-five males and fourteen females) from the Lithuanian junior rowing national team were recruited for the study using a purposive sampling approach. All eligible athletes who were available during the study period were invited to participate. To be included in the study, the rowers were required to have at least five years of training experience, to be members of the national team for their age group, and to be free from acute illness or injury at the time of testing. The participants ranked among the top ten in their respective age groups at the European Championships, which reflects the high competitive level of the sample. All participants were Caucasian. The descriptive characteristics of the participants are presented in Table 1.

### 2.2. Study Design

**Figure 1:**
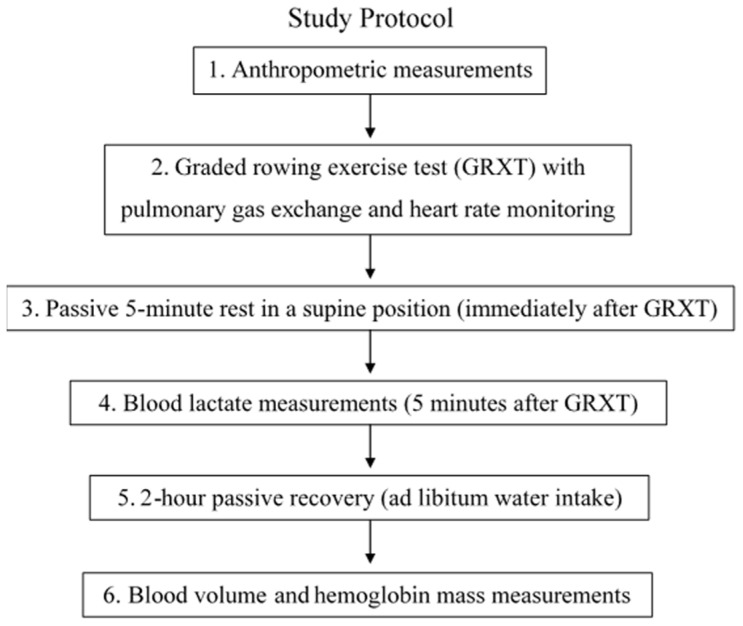
Flowchart of the study protocol.

Before testing, participants followed standardized pre-test instructions, including consumption of a standardized meal, abstention from alcohol, refraining from caffeine for at least 3 h, arriving fully hydrated, and avoiding high training volume or high-intensity exercise for at least 24 h before the laboratory visit. Individual testing schedules were created after the participants provided written consent. The participants had to visit the laboratory once. Upon arrival, a bioimpedance measurement was performed before the graded rowing exercise test (GRXT). The GRXT was then conducted to determine V̇O_2_max and maximum heart rate (HRmax). Total BV and Hb-mass measurements were performed two hours after completing the GRXT. During the 2-h period following the GRXT, participants were asked to drink water ad libitum. Hydration intake during this interval was not standardized or quantified. The study protocol is presented in a flowchart (Figure 1).

### 2.3. Data Collection and Analysis

#### 2.3.1. Anthropometry

Anthropometric data, such as BM, FM (%), and FFM (kg), were determined using body composition analyzer TBF–300 (TANITA, Tokyo, Japan), which employs the principals of bioelectrical impedance analysis.

#### 2.3.2. Pulmonary Gas Exchange

Pulmonary gas exchange variables were measured on a breath-by-breath basis and averaged every 5 s during the graded rowing exercise test (GRXT) and recovery periods via the portable pulmonary gas exchange analyzer MetaMax 3B (Cortex, Leipzig, Germany) and the “Hans Rudolph” face mask. A portable gas exchange analyzer was shown to provide reliable measurements of metabolic demand with adequate validity for field-based measurements [20]. Heart rate (HR) was also continuously measured and averaged every 5 s throughout the GRXT and recovery periods. The pulmonary gas exchange analyzer was calibrated according to the manufacturer’s recommendations before each test. Each peak value was the peak 20-s interval average value attained during the GRXT.

#### 2.3.3. Graded Rowing Exercise Test

The participants completed a gradually increasing workload on an air braked rowing ergometer Concept 2 (Model D, Concept 2, Inc., Morrisville, VT, USA), with the drag factor adjusted individually. The participants completed a 10-min warm-up according to their preferences. The initial workload was selected according to participant sex, reflecting known sex-related differences in rowing performance capacity. No additional adjustment for body mass or training level was applied because all participants were elite junior rowers with a relatively homogeneous training background. The GRXT started at a 2-min workload of 150 W for males and 100 W for females. The workload then increased by 25 W every 30 s. The test was terminated when the participant could no longer maintain the necessary power output or until exhaustion.

#### 2.3.4. Blood Lactate Measurements

After performing the GRXT, the participants had to rest in a supine position for 5 min, after which a capillary blood sample was taken to measure the blood lactate concentration ([La]5′) via the Lactate Pro 2 (Arkray, Kyoto, Japan) analyzer.

#### 2.3.5. Blood Volume and Haemoglobin Mass Measurement

Following a 2-h passive recovery period after the test, participants rested for an additional 15–20 min in the supine position. During this time, the palm and fingers of the left hand were warmed using a specialized electrically heated glove. Before each measurement, the breathing circuit was carefully assembled according to the manufacturer’s instructions. To ensure the accuracy and reliability of the results, all necessary equipment checks were performed before tests. A “leak test” was carried out according to the instructions of the manufacturers to automatically monitor the system for any leaks, which is an essential step to confirm that the circuit is airtight, as even a small leak from an incorrectly assembled component can cause loss of carbon monoxide and lead to overestimation of blood volume measurements. After the successful “leak test”, three arterialized blood samples (100 μL each) were taken from the prewarmed fingertip. The three samples were immediately analyzed via a blood gas analyzer (ABL80, Radiometer, Copenhagen, Denmark) to determine the haemoglobin concentration ([Hb]), haematocrit (Hct), and percentage of carboxyhaemoglobin (%HbCO). After that, the participants were connected to a semiautomated system (Detalo Performance^TM^, Detalo Health ApS, Hørsholm, Denmark.) and breathed 100% O_2_ (Linde GmbH, Pullach, Germany.) for 1 min to flush nitrogen from their respiratory system. They then continued breathing a mixture of O_2_ and 99.5% chemically pure carbon monoxide (CO) (Linde GmbH, Pullach, Germany.)—1.0 mL/kg for males and 0.8 mL/kg for females—for another 6 min via a mouthpiece. When 3 min passed after the rebreathing, another three arterialized blood samples (100 μL each) were taken and analyzed. Using a CO rebreathing technique integrated into a semiautomated system, we measured the change in %HbCO to calculate Hb-mass, considering the small amount of CO that remained in the rebreathing circuit at the end of the procedure, which was measured using a CO detector (Drägerwerk AG & Co. KGaA, Lübeck, Germany) [21,22,23]. Notably, the typical measurement error for such blood volume measurement method is very low (TE ≤ 1.2%) [24,25,26]. The total Hb-mass, red blood cell volume (RBCV), plasma volume (PV), and BV were calculated using a dilution principle, where the amount of CO administered, unused, and bound to Hb, is known, and then calculated from Hct, [Hb], and the fraction of %HbCO [22,23].

#### 2.3.6. Statistical Analysis

The data provided in the tables and graphs are presented as the means and standard deviations. The Shapiro–Wilk test was conducted to assess the normality of distribution. ANOVA and effect size were calculated to assess the differences between sexes. The effect size was calculated using Cohen’s d method by dividing the two population mean differences by the pooled standard deviation. Pearson’s correlation coefficient was calculated to assess the relationships among variables. The correlation coefficients from 0.30–0.50 (or from −0.30–0.50) were considered low, those from 0.50–0.70 (or from −0.50–0.70) were considered moderate, those from 0.70–0.90 (or from −0.70–0.90) were considered high, and those from 0.90–1.00 (or from −0.90–1.00) were considered very high [27]. The differences and correlations were considered statistically significant at *p* < 0.05. All calculations were performed via the Statistical Package for Social Sciences (SPSS v.27, SPSS Inc., Chicago, IL, USA).

## 3. Results

The absolute and BM-relative power and oxygen uptake values during GRXT were greater in the male rowers than in the female rowers. However, when expressed relative to FFM, power and oxygen uptake values, as well as HRmax and [La]5′, did not differ between sexes (Table 2).

The [Hb], as well as absolute and BM-relative Hb-mass and RBCV values, were greater in male rowers than in female rowers. However, the Hb-mass relative to FFM and the RBCV relative to FFM did not differ between the sexes. The absolute PV was greater in male rowers, whereas the PV relative to FFM was greater in female rowers. PV relative to BM did not differ between sexes. The absolute and BM-relative BV were greater in male rowers, but the BV relative to FFM did not differ between sexes (Table 3).

Among all the correlations between BM and haematological parameters, only FFM was significantly correlated with total Hb-mass in male rowers. In contrast, BM and FFM in females were strongly and positively correlated only with absolute PV and BV (Table 4).

Among all correlations between maximal power values during GRXT and hematological parameters, only absolute Hb-mass correlated with absolute peak power (r = 0.418, *p* < 0.05) in male junior rowers. In contrast, only the relative Hb-mass, PV, and BV values were correlated with the relative maximal power values in female junior rowers (Table 5).

**Figure 2:**
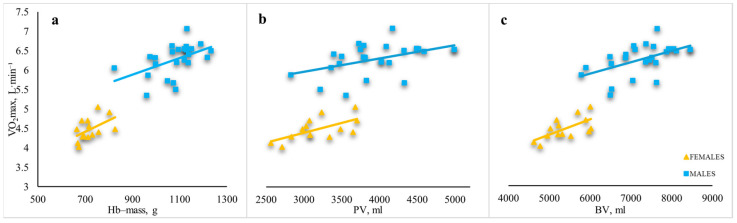
Relationships between absolute V̇O_2_max and absolute haematological parameters in male and female junior rowers. (**a**) relationships between absolute V̇O_2_max and absolute Hb-mass; (**b**) relationships between absolute V̇O_2_max and absolute PV; (**c**) relationships between absolute V̇O_2_max and absolute BV. Note: Hb-mass—haemoglobin mass; PV—plasma volume; BV—blood volume; V̇O_2_max—maximum oxygen uptake.

The relationships between the absolute V̇O_2_max and total Hb-mass was similar in females (r = 0.497, *p* < 0.01) and males (r = 0.487, *p* < 0.01) (Figure 2a). The relationship between the absolute V̇O_2_max and PV was stronger in females (r = 0.634, *p* < 0.01) than in males (r = 0.457, *p* < 0.01) (Figure 2b). The relationship between the absolute V̇O_2_max and BV was stronger in females (r = 0.646, *p* < 0.05) than in males (r = 0.530, *p* < 0.01) (Figure 2c).

**Figure 3:**
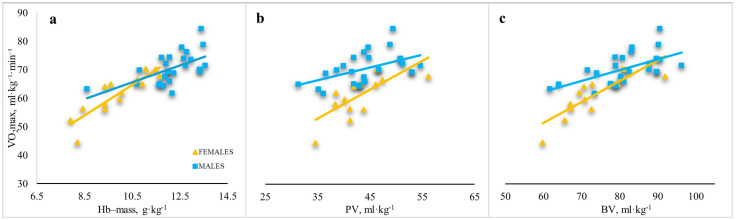
Relationship between BM relative V̇O_2_max and BM relative hematological parameters in male and female junior rowers. (**a**) relationships between BM relative V̇O_2_max and BM relative Hb-mass; (**b**) relationships between BM relative V̇O_2_max and BM relative PV; (**c**) relationships between BM relative V̇O_2_max and BM relative BV. Note: Hb-mass—haemoglobin mass; PV—plasma volume; BV—blood volume; V̇O_2_max—maximum oxygen uptake.

The relationships between BM-relative V̇O_2_max and BM-relative Hb-mass was stronger in females (r = 0.875, *p* < 0.01) than in males (r = 0.580, *p* < 0.01) (Figure 3a). The relationship between BM-relative V̇O_2_max and BM-relative PV was stronger in females (r = 0.725, *p* < 0.01) than in males (r = 0.504, *p* < 0.01) (Figure 3b). The relationship between BM-relative V̇O_2_max and BM-relative BV was stronger in females (r = 0.837, *p* < 0.01) than in males (r = 0.593, *p* < 0.01) (Figure 3c).

**Figure 4:**
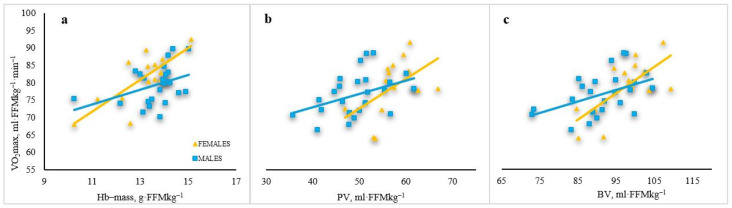
Relationships between FFM relative V̇O_2_max and FFM relative hematological parameters in male and female junior rowers. (**a**) relationships between FFM relative V̇O_2_max and FFM relative Hb-mass; (**b**) relationships between FFM relative V̇O_2_max and FFM relative PV; (**c**) relationships between FFM relative V̇O_2_max and FFM relative BV. Note: Hb-mass—haemoglobin mass; PV—plasma volume; BV—blood volume; V̇O_2_max—maximum oxygen uptake; FFM—fat-free mass.

The relationship between FFM-relative V̇O_2_max and FFM-relative total Hb-mass was stronger in females (r = 0.771, *p* < 0.01) than in males (r = 0.383, *p* < 0.05) (Figure 4a). The relationship between FFM-relative V̇O_2_max and FFM-relative PV was stronger in females (r = 0.502, *p* < 0.01) than in males (r = 0.417, *p* < 0.05) (Figure 4b). The relationship between FFM-relative V̇O_2_max and FFM-relative BV was stronger in females (r = 0.697, *p* < 0.01) than in males (r = 0.462, *p* < 0.05) (Figure 4c).

## 4. Discussion

This study is the first to describe BV and Hb-mass, aerobic capacity, and their interrelationships, as well as their relationships with BM and FFM in elite male and female junior rowers. The findings demonstrate sex-based differences in haematological and aerobic capacity variables among elite junior rowers. The absolute and BM-normalized V̇O_2_max, peak power, total BV, and Hb-mass are greater in males; however, these differences disappear when adjusted for FFM, suggesting that body composition plays a key role in determining these parameters. Interestingly, the PV relative to FFM is greater in female rowers. Furthermore, the correlations between hematological, anthropometric, and submaximal/maximal power variables obtained during the GRXT differ between sexes, with PV and BV correlating with both FFM and BM in females, whereas in males, only Hb-mass correlates with FFM. In addition, in females, relative Hb-mass, PV, and BV are significantly correlated with relative peak power values, whereas in males, absolute Hb-mass is correlated with only absolute maximal power values during the GRXT. Notably, BV, PV and Hb-mass correlated with V̇O_2_max in both sexes, although stronger associations were observed in female junior rowers.

### 4.1. Anthropometric Characteristics of Junior Elite Rowers

The anthropometric characteristics of the rowers in this study are consistent with those reported for national- and international-level athletes, with some distinctions by sex and performance level. World-class open-category rowers typically stand 194 ± 5 cm (men) and 181 ± 5 cm (women) tall and exhibit a mesomorphic somatotype, with BM values of 94 ± 6 kg for men and 76 ± 5 kg for women [28]. The male rowers in this study (18.4 ± 2.4 years; 1.92 ± 0.05 m; 89.3 ± 4.7 kg; 11.2 ± 3.4% FM; 72.1 ± 3.6 kg FFM) closely resemble elite junior and young senior athletes reported by Mikulić et al. [5] and Blervaque et al. [29], but fall below elite senior values for height (194–198 cm), BM (93.8–97.7 kg), and FFM (>81 kg) [5,10,11]. The slightly lower BM and FFM observed in our junior athletes likely reflect age and training differences, which influence muscle mass and strength development. Nevertheless, their height and lean body composition indicate strong potential for achieving elite performance.

The height and BM of the female rowers in this study (17.0 ± 1.9 years; 1.77 ± 0.07 m; 74.2 ± 11.3 kg; 24.5 ± 5.8% FM; 55.7 ± 6.7 kg FFM) were similar to those of the elite female rowers reported by Bourdin et al. [30] and Tran et al. [31], although the cohort studied by Tran et al. was significantly older. Compared with younger elite female rowers [32], the athletes in the present study presented greater FFM and FM, possibly due to age-related factors. Notably, their FM exceeded typical elite values (18–22%) reported in earlier studies [18,33].

In our study, male rowers were significantly taller, heavier, and leaner than female rowers were, with higher FFM and lower FM; this trend has been consistently reported across multiple studies [6,34,35]. Overall, both male and female athletes display anthropometric profiles compatible with high-level rowing, particularly given their young age, although slight variations in body composition—especially higher FM in females—may warrant attention for optimizing performance potential.

### 4.2. Aerobic Capacity (V̇O_2_max) in Junior Rowers

The absolute V̇O_2_max of our male athletes (6.278 ± 0.400 L/min) falls within the elite and international range (5.7–6.3 L/min) [5,10,31,33], with relative values commonly exceeding 64 mL/kg/min [11,29]. Our youth rowers exceeded this value, averaging 70.17 ± 5.58 mL/kg/min, aligning with U17–U19 rowers (70.0 ± 5.9 mL/kg/min) [34].

The female athletes presented an absolute V̇O_2_max of 4.478 ± 0.288 L/min, similar to that of elite rowers (4.5 L/min) [31] and higher than the values reported by Bourdin et al. [30] and Gore et al. [18] (3.78–3.80 L/min). Their relative V̇O_2_max (61.28 ± 7.28 mL/kg/min) surpassed that of national-level Polish athletes (52–55 mL/kg/min) [6] and collegiate rowers (55.2 mL/kg/min) [36] and was above the 49–50 mL/kg/min reported in international-level youth rowers [32].

V̇O_2_max progression is evident with increased training experience and age, as shown by Mikulić et al. [5], where elite senior males presented higher V̇O_2_max (5.67 ± 0.27 L/min) than their junior and sub-elite senior counterparts did. Although our junior rowers presented absolute V̇O_2_max values similar to those of adult elite rowers, the latter likely possess greater anaerobic capacity, superior rowing technique, and longer training experience, allowing them to cover race distances more efficiently despite comparable V̇O_2_max levels.

### 4.3. Hematological Characteristics: Hb-Mass, Blood Volume, and Plasma Volume

The male junior rowers in our study had a mean Hb-mass of 1082.6 ± 90.7 g, exceeding that of non-athletes (901 ± 123 g) [37] and physically active individuals (1003 ± 145 g) [38], similar to other rowers (1025 ± 88 g) [13], but below that of elite individuals (1258 ± 123 g) and world-class rowers (1367 ± 130 g) [10,11]. Relative to BM (12.17 ± 1.07 g/kg) and FFM (13.68 ± 0.96 g/kg FFM), these values exceeded those of non-athletes (11.5 ± 1.5 and 14.9 ± 1.6) [37], were slightly lower than those of physically active individuals (13.9 ± 1.5 and 16.1 ± 1.7) [38], matched those of other rowers (12.3 ± 0.8 and 15.4 ± 1.2) [13,18], and remained lower than elite/world-class standards (13.7–14.0 and 15.8 ± 0.7) [10,11].

Compared with males, female rowers presented a lower absolute Hb-mass (720.2 ± 49.3 g), but the Hb-mass exceeded that of non-athletes (595 ± 91 g) [37] and physically active individuals (641 ± 84 g) [38] and matched that of other rowers (698 ± 63 g) [13]. Relative to BM (9.86 ± 1.20 g/kg) and FFM (13.04 ± 1.21 g/kg FFM), the values surpassed those of non-athletes (8.9 ± 1.4 and 14.0 ± 1.5) [37], were similar to those of physically active individuals (10.3 ± 1.0 and 13.6 ± 1.3) [38], and were close to those of other rowers (9.4 ± 0.9 and 13.3 ± 0.7) [13].

The total BV of male rowers was 7270.0 ± 716.8 mL, with relative values of 81.62 ± 8.44 mL/kg and 91.92 ± 8.39 mL/kg FFM. The total BV of non-athletes (6279 ± 829 mL) [37] and physically active individuals (6783 ± 903 mL) [38] was similar to that of other rowers (7528 ± 1343 mL) [13] but lower than that of world-class athletes (9759 ± 1092 mL) [10] and 9025 ± 1130 mL [11]. Relative to BM, male BV was comparable to that of nonathletes (80.3 ± 10.8 mL/kg) [37], lower than that of active individuals (93.7 ± 8.7 mL/kg) [38], similar to that of rowers (81.9 ± 5.1 mL/kg) [13], and lower than that of world-class rowers (100 ± 7 mL/kg) [10]. Compared with those in FFM, the BV in males is lower than that in non-athletes (105.3 ± 11.2 mL/kg) [37], physically active individuals (108.7 ± 9.0 mL/kg) [38], and rowers (102.7 ± 8.3 mL/kg) [13].

The total BV of females (5388.1 ± 470.8 mL) exceeded that of non-athletes (4682 ± 738 mL) [37] and active individuals (4983 ± 640 mL) [38] and was similar to that of rowers (5151 ± 218 mL) [13]. BV relative to BM in females (73.51 ± 8.34 mL/kg), matched non-athletes (70.3 ± 11.3 mL/kg) [37] and rowers (70.0 ± 4.8 mL/kg) [13] but was lower than that in physically active individuals (80.1 ± 6.9 mL/kg) [38]. BV relative to FFM (97.35 ± 7.57 mL/kg) was lower than that in non-athletes (110.9 ± 12.5 mL/kg) [37] and physically active individuals (105.3 ± 8.4 mL/kg) [38], but close to values reported in rowers (99.2 ± 5.1 mL/kg) [13].

The PV of males (3948.7 ± 531.9 mL) exceeded that of non-athletes (3570 ± 510 mL) [37] and was similar to that reported for rowers (4182 ± 828 mL) [13]. Relative to BM, male values (44.32 ± 6.12 mL/kg) aligned with non-athletes (45.7 ± 6.9 mL/kg) [37] and rowers (44.2 ± 4.2 mL/kg) [13]. Compared with FFM (49.94 ± 6.53 mL/kgFFM), male PV was lower than that in non-athletes (60.2 ± 7.7 mL/kg) and rowers (55.4 ± 6.3 mL/kg).

Compared with male rowers, female rowers presented a lower absolute PV (3174.3 ± 359.4 mL) but relatively high values when adjusted for BM (43.24 ± 5.24 mL/kg) and especially FFM (57.23 ± 4.79 mL/kg), indicating efficient plasma expansion. The PV of females was greater than that of non-athletes (2861 ± 480 mL) [37] and comparable to that of rowers (3014 ± 98 mL) [13]. Relative to BM, female PV matched non-athletes (43.0 ± 7.2 mL/kg) and slightly exceeded values in rowers (41.2 ± 2.5 mL/kg). Compared with those of non-athletes, the values of FFM were lower (67.7 ± 8.6 mL/kg) but close to those of rowers (58.5 ± 4.1 mL/kg) [13,37].

While the observed differences in Hb-mass, BV, and PV are partially related to FFM, since more lean tissue is required and supports greater oxygen delivery capacity, other key factors also contribute. These include training-induced cardiovascular adaptations, such as increased erythropoiesis (stimulated by hypoxia and training volume), hormonal differences (e.g., testosterone in males), and altitude exposure or training history. Elite adult athletes often have higher absolute and relative BV not only because of greater muscle mass but also because of longer training durations, higher overall workloads, and greater plasma expansion capacity over time.

### 4.4. Allometric Scaling and Interpretation of Relative Physiological Variables

Rowers, despite possessing some of the highest absolute values for aerobic capacity, BV, and Hb-mass among all athletes, typically display lower values for these variables when expressed relative to BM or FFM—a pattern also observed in the general untrained population, who exhibit similar relative values despite much lower absolute physiological capacity. This phenomenon is best explained by allometric scaling, where aerobic and hematological parameters increase with body size according to a power–law relationship with a scaling exponent less than one (generally 0.67–0.75 for aerobic capacity), resulting in disproportionally lower relative values as the body or FFM increases [39,40,41]. Compared with those of other endurance athletes, the greater BM and FFM of rowers amplify this effect, causing their relative values to approach those of untrained individuals of similar size, even though their absolute capacities remain far superior. This convergence in relative values between elite rowers and untrained people with similar body compositions underscores the importance of considering allometric relationships when interpreting physiological data, as relying solely on relative values may underestimate the exceptional absolute capacities and training adaptations present in larger athletes such as rowers.

### 4.5. Sex Differences in Hematological Parameters

In our study, sex differences in absolute Hb-mass (50.3%) exceeded those reported in non-athletes (34%) [37] but were smaller than those reported in active individuals (56.5%) [38] and rowers (56.8%) [13]. Relative to BM, the gap (23.4%) matched non-athletes (23%), yet was below active (35%) and rowing cohorts (30.1%). When adjusted for FFM, the difference (4.9%) was close to that of non-athletes (6%) but far smaller than that of active persons (18.4%) or rowers (15.8%). The absolute BV differences (34.9%) were greater for non-athletes (25%), similar to those for active individuals (36%), and smaller than those for rowers (46.2%). BV relative to BM (11%) was comparable to that of non-athletes (13%) but lower than that of the active (17%) and rowing groups (17%); relative to FFM (–5.69%), it matched that of non-athletes (–6%) and was slightly greater than that of the active (–3.2%) and rowing groups (–3.5%). The absolute PV differences (24.4%) were slightly greater for non-athletes (20%) than for rowers (38.8%). PV relative to BM (2.5%) was lower than that in non-athletes (6%) and rowers (7.3%); relative to FFM (–12.7%), PV relative to BM was similar to that in non-athletes (–11%) but exceeded the gap in rowers (–5.3%).

As in previous studies, sex differences in absolute blood variables were greater than those in BM- or FFM-normalized values. Compared with FFM females, females presented higher or similar BV and especially PV values. Our findings align with those of Kontro et al. [42], who reported that despite lower absolute RBCV and Hb_masss_, females had higher BV (+4%) and PV (+14%) per FFM, indicating relative plasma volume expansion. BV correlated strongly with FFM, not total BM, with men’s greater FFM explaining greater absolute BV and RBCV; FFM was the main predictor of BV and Hb_mass_, whereas FM had a minimal effect [42]. Testosterone promotes erythropoiesis via increased erythropoietin, reduced hepcidin, and direct stimulation of red cell production, increasing RBC mass and Hb levels in males [43,44]. A lower Hct means that a given FFM corresponds to a larger plasma compartment, yielding a higher PV but a lower RBCV per FFM [42,45]. Estrogen lowers osmotic thresholds for vasopressin release and thirst, promoting antidiuresis and plasma expansion, particularly in the mid-luteal phase [46,47]. It also modulates the RAAS by suppressing the vasoconstrictor axis (ACE/Ang II/AT_1_) and enhancing the vasodilator–natriuretic arm (ACE2/Ang–(1–7)/Mas, AT_2_), whereas progesterone antagonizes MR, reducing aldosterone-driven sodium retention. Together, these hormonal effects favor higher PV per FFM in women [48,49,50].

### 4.6. Role of Fat-Free Mass in Oxygen Transport Capacity

The FFM is a critical determinant of BV and total Hb-mass in elite athletes [51]. In our study, the correlations between the anthropometric and hematological variables differed between the sexes. Only FFM was significantly associated with total Hb-mass in male rowers. Similarly, in elite male rowers, a strong correlation was observed between total Hb-mass and BM (r = 0.94) and between total Hb-mass and LBM (r = 0.92) [11]. In contrast, in our female rowers, both BM and FFM were strongly and positively correlated only with absolute PV and BV. In contrast, in non-athletic adults, there was a strong positive correlation between lean BM and BV (R^2^ = 0.71, *p* < 0.001) as well as LBM and Hb-mass (R^2^ = 0.79, *p* < 0.001), and this association was minimally affected by adjustment for sex [37]. Similarly, Faltz et al. [38] reported no statistically significant sex differences in correlation coefficients between LBM and Hb-mass.

Therefore, our findings contribute to a growing body of literature that demonstrates a robust association between FFM and indices of oxygen transport capacity, including Hb-mass, RBCV, and total BV. In both the general population and athletes, muscle tissue, the predominant component of FFM, serves as the primary consumer of oxygen during exercise. Consequently, individuals with greater FFM exhibit heightened oxygen demands not only at rest but also during physical activity. This increased demand elicits adaptations in the hematological system, notably an expansion in total BV and Hb-mass, to optimize oxygen delivery to working muscles [39,52].

A key mechanism linking FFM to hematological parameters is the stimulation of erythropoiesis. Increases in muscle mass, whether through development or training, increase the body’s need for oxygen, thereby increasing erythropoietic activity through hypoxia-inducible pathways and increasing erythropoietin (EPO) secretion [53]. This adaptive response leads to increases in both the RBC count and [Hb]. However, in endurance-trained populations, such as rowers, plasma volume expansion often exceeds the increase in RBC mass. This phenomenon, known as “sports anaemia,” results in a dilutional decrease in [Hb], although total Hb-mass remains elevated compared with that of untrained individuals [39].

Rowers in particular exemplify these adaptations owing to the sport’s unique physiological demands. Rowing is characterized by high muscle mass involvement, high-intensity efforts, and significant aerobic and anaerobic contributions. As a result, rowers consistently demonstrate higher FFM, greater BV, and increased Hb-mass relative to both non-athletes and athletes from less-demanding disciplines [54]. The positive correlation between FFM and haematological indices in rowers has been well documented, with evidence suggesting that these adaptations directly contribute to enhanced aerobic performance and elevated V̇O_2_max [52].

In summary, the interplay between FFM and haematological parameters reflects an integrated physiological adaptation to increased muscle mass and physical activity. In rowers, these mechanisms are particularly pronounced, underpinning their exceptional aerobic and power capacities.

### 4.7. Hematological Determinants of Aerobic Capacity

In our study, all measured hematological variables exhibited robust correlations with V̇O_2_max in both sexes, with stronger associations observed in female junior rowers. These findings are consistent with previous studies highlighting the central role of haematological status in determining aerobic performance [13,18,55]. For example, Gore et al. [18] reported that the correlation between relative Hb-mass and V̇O_2_max was especially pronounced in female rowers (r = 0.92) compared with that in male rowers (r = 0.79) and male runners (r = 0.48). Similarly, a comprehensive meta-analysis by Webb et al. [55] across 384 studies confirmed a positive association between V̇O_2_max and various haemoglobin metrics (concentration, mass, and haematocrit) irrespective of sex. However, the present study revealed sex-specific patterns in maximal power correlations, which might reflect physiological or maturational differences in adolescent athletes—a topic underexplored in previous large-scale meta-analyses that often lack sufficient female representation.

Our results corroborate those of Lundby et al. [13], who reported a strong pretraining association between Hb-mass and VO_2_peak in both male and female rowers. However, this association weakened after lean body mass was adjusted for, suggesting that while Hb-mass is a critical determinant of aerobic capacity, its functional significance is partly mediated by an athlete’s body composition. This interplay aligns with prior research indicating that hematological parameters such as Hb-mass and BV are typically elevated in endurance-trained athletes and are fundamental to their superior aerobic capacity [39,51].

### 4.8. Mechanisms, Genetic Influences, and Sex-Specific Adaptations

Mechanistically, these associations are well understood; total Hb-mass enhances the oxygen-carrying capacity of the blood, directly impacting V̇O_2_max [11,16,22]. Moreover, increases in BV, including PV and red cell volume, support cardiovascular function by maintaining stroke volume and effective O_2_ transport via the Frank–Starling mechanism. Interventional studies have demonstrated that artificially augmenting red cell mass through erythropoietin or reducing it via phlebotomy modulates V̇O_2_max accordingly [22]. These findings underscore the functional importance of haematological adaptation to endurance training, both in terms of O_2_-carrying capacity and the cardiovascular adaptations that support it [56].

Genetic factors may also contribute to individual variability in haematological adaptation and aerobic performance. Recent studies have identified the NFIA–AS2 rs1572312:C>A polymorphism as a potential determinant of Hb-mass and aerobic indices in endurance athletes [57]. These findings open avenues for further exploration of genetic predispositions that may explain the interindividual differences observed in the present study, especially given the sex-specific trends in hematological–aerobic correlations.

Overall, the present findings support the critical role of hematological parameters, particularly hemoglobin mass (Hb-mass) and blood volume, in determining aerobic capacity among elite young rowers. Notably, these associations were more pronounced in female athletes, suggesting a potentially greater reliance on hematological adaptations within this group. One possible explanation is that elite female rowers may operate closer to their maximal capacity for peripheral oxygen extraction, thereby requiring further improvements in oxygen delivery to enhance maximal oxygen uptake (VO_2_max). In contrast, male athletes may retain a greater capacity to augment both oxygen delivery and extraction mechanisms.

Additionally, nutritional factors may contribute to these observed sex differences. For instance, the higher prevalence of iron deficiency among female athletes could constrain increases in Hb-mass, thereby representing a limiting factor for aerobic performance development.

The current findings also emphasize the importance of considering both absolute and relative measures of power and aerobic indices, as well as adjusting for confounders such as FFM when these associations are interpreted.

Future research should aim to elucidate the mechanistic basis of the observed sex differences and incorporate larger, sex-stratified cohorts to confirm these trends. Furthermore, the integration of genetic profiling may help clarify the interplay between heritable factors and hematological adaptations in shaping aerobic performance in young athletes.

### 4.9. Practical Applications for Training and Talent Development

The mean 2000 m competitive times of the studied athletes (377.8 ± 8.2 s in males and 434.8 ± 11.5 s in females) indicate the general performance level of the cohort. However, these results should be interpreted with caution, as competitive times in rowing are influenced not only by physiological capacity but also by boat class, environmental conditions, weather, and race context. Therefore, in the present study, competitive performance data were used only to describe the sporting level of the athletes and not for formal correlation analysis with aerobic capacity or hematological variables. Consequently, any relationship between higher sporting level and the studied physiological parameters can only be discussed theoretically and in light of previous literature. The present findings are most directly applicable to highly trained junior athletes and should not be directly generalized to elite senior or recreational rowers. The results of the present study can help individualize training strategies in elite junior rowers by highlighting the role of sex-specific physiological profiles in aerobic performance. Coaches and sports scientists may use such data to modify training loads, monitor adaptations, and optimize recovery protocols according to differences in BV, Hb-mass, and their relationship with aerobic capacity. The findings also provide a physiological reference for talent identification and progression monitoring in youth rowing. In addition, the observed patterns may serve as a basis for further research in other endurance-based sports, helping to refine sport-specific conditioning approaches and to better understand sex differences in oxygen transport and utilization.

### 4.10. Study Limitations

This study has several limitations that should be considered when interpreting the results. First, the present study used a single-time cross-sectional design; therefore, the results represent only a snapshot and do not reflect possible seasonal or training-state variability. However, this approach was appropriate for the specific aims of the study, which did not require longitudinal monitoring. Future studies should examine these parameters repeatedly across different phases of the training and competitive season. Second, although all the athletes were tested in season, potential confounding factors such as training load, nutritional status, and hydration level were not controlled for. Third, while the observed correlations between blood parameters and V̇O_2_max provide valuable insight, they do not confirm a direct mechanistic link, as other determinants of aerobic capacity (e.g., maximal cardiac output and muscle oxygenation) were not evaluated. Fourth, the hydration status was not formally monitored after the GRXT. Although participants were allowed to drink water ad libitum during the 2-h recovery period, fluid intake was not quantified. Therefore, some residual influence of post-exercise changes in plasma volume on hematological measurements cannot be completely excluded. Nevertheless, the 2-h interval between exercise cessation and blood-related measurements likely reduced the most immediate acute effects of exercise on these variables. Fifth, this study was conducted in a relatively homogeneous sample comprising only Caucasian athletes from the Lithuanian junior national team. Therefore, the findings are most directly applicable to athletes with similar demographic and training characteristics. Caution is warranted when extrapolating these results to other ethnic or geographic populations, as hematological variables related to oxygen transport, and potentially the determinants of aerobic capacity, may differ across populations due to genetic and environmental influences, such as ambient temperature or atmospheric pressure. Sixth, body composition was measured using the TANITA TBF-300 analyzer, which applies bioelectrical impedance analysis. While this method is practical and suitable for routine assessment, it is less precise than gold-standard methods such as DXA or hydrostatic weighing. Moreover, because bioelectrical impedance is sensitive to hydration status and body fluid distribution, the obtained estimates of fat mass and fat-free mass may have been affected by acute physiological variations at the time of measurement. Consequently, the body composition results should be interpreted with caution. Finally, the relatively small and unequal sample size between sexes, particularly the smaller female group, may have reduced the robustness of the between-sex comparisons and limited the ability to detect smaller effects, especially in fat-free-mass-normalized variables. The correlations do not imply causation, and other unexamined variables might influence the results. Deeper investigations are needed to understand the causes of differences between the sexes.

## 5. Conclusions

Absolute and relative to BM total BV, Hb-mass and aerobic capacity variables are greater in elite junior male rowers than in females but are similar when adjusted for FFM. In contrast, PV relative to FFM is greater in elite junior female rowers than in males.

Correlations among haematological and anthropometric parameters, as well as among haematological and maximal power values, differ between male and female junior rowers. PV and BV are correlated with both FFM and BM in female junior rowers, whereas only Hb-mass is correlated with FFM in male junior rowers.

Relative Hb-mass, relative PV and relative BV correlate with relative power values in female junior rowers; in contrast, Hb-mass correlates only with absolute power in male junior rowers.

All haematological variables correlate with V̇O_2_max in both male and female junior rowers, although higher correlations are observed in females.

## Figures and Tables

**Table 1:** Characteristics of the participants.

	Age (Years)	Height (m)	BM (kg)	BSA (m^2^)	FM (%)	FFM (kg)	2000-m (s)
MALES (*n* = 25)	18.4 ± 2.4 (15.0–22.0)	1.92 ± 0.5 (1.84–2.03)	89.3 ± 4.71 (79.3–96.3)	2.19 ± 0.08 (2.04–2.35)	11.2 ± 3.4 (3.6–16)	72.1 ± 3.63 (71.1–86.5)	377.8 ± 8.2 (367.1–397.0)
FEMALES (*n* = 14)	17.0 ± 1.9 (14.0–22.0)	1.77 ± 0.7 (1.66–1.94)	74.2 ± 11.3 (64.8–100.7)	1.91 ± 0.17 (1.72–2.32)	24.5 ± 5.8 (16.0–34.8)	55.7 ± 6.67 (48.6–69.2)	434.8 ± 11.5 (419.0–453.2)
*p*	0.073	0.000	0.000	0.000	0.000	0.000	0.000

Note: BM—body mass; BSA—body surface area; FM—fat mass; FFM—fat-free mass. The ranges are presented in the parentheses.

**Table 2:** Submaximal and maximal values of power and oxygen uptake during GRXT in male and female rowers.

	Males (*n* = 25)	Females (*n* = 14)	*p*	Effect Size
Peak power, W	487.0 ± 33.9 (425.0–550.0)	337.5 ± 23.5 (300.0–375.0)	0.000	4.872
Peak power, W·kg^−1^	5.47 ± 0.43 (4.44–6.34)	4.64 ± 0.74 (3.23–5.77)	0.000	1.469
Peak power, W·FFMkg^−1^	6.16 ± 0.41 (5.29–6.93)	6.13 ± 0.76 (4.70–7.37)	0.887	0.048
HRmax, bpm	192.4 ± 7.77 (175.0–205.0)	189.00 ± 8.96 (177.0–206.0)	0.222	0.414
V̇O_2_max, L·min^−1^	6.278 ± 0.400 (5.350–7.070)	4.478 ± 0.288 (4.020–5.050)	0.000	4.943
V̇O_2_max, mL·kg^−1^·min^−1^	70.17 ± 5.58 (61.70–84.40)	61.28 ± 7.28 (44.50–70.30)	0.000	1.505
V̇O_2_max, mL·FFMkg^−1^·min^−1^	79.43 ± 5.31 (70.11–89.74)	81.12 ± 7.25 (67.99–92.48)	0.411	–0.278
[La]5′, mmol·L^−1^	14.37 ± 3.55 (9.9–21.5)	12.32 ± 4.31 (6.7–24.1)	0.071	0.620

Note: FFM—fat-free mass; HRmax—maximum heart rate; V̇O_2_max—maximum oxygen uptake; [La]5′—blood lactate concentration 5 min after the graded rowing exercise test. The ranges of the values are presented in parentheses.

**Table 3:** Hematological parameters of female and male rowers.

	Males (*n* = 25)	Females (*n* = 14)	*p*	Effect Size
[Hb], g·L^−1^	149.5 ± 9.5 (133.4–167.0)	134.1 ± 6.9 (120.6–145.0)	0.000	1.763
Hct, %	45.8 ± 2.9 (41.0–51.1)	41.2 ± 2.1 (37.2–44.5)	0.000	1.776
Hb-mass, g	1082.6 ± 90.7 (824.0–1233.0)	720.2 ± 49.3 (664.0–826.0)	0.000	4.607
Hb-mass, g·kg^−1^	12.17 ± 1.07 (8.60–13.50)	9.86 ± 1.20 (7.90–11.60)	0.000	2.065
Hb-mass, g·FFMkg^−1^	13.68 ± 0.96 (10.3–15.0)	13.04 ± 1.21 (10.3–15.1)	0.076	0.609
RBCV, mL	3320.7 ± 277.6 (2537.0–3786.0)	2213.4 ± 151.1 (2042.0–2537.0)	0.000	4.597
RBCV, mL·kg^−1^	37.28 ± 3.29 (26.5–41.6)	30.29 ± 3.64 (24.4–35.7)	0.000	2.049
RBCV, mL·FFMkg^−1^	41.97 ± 2.95 (31.6–46.1)	40.09 ± 3.72 (31.6–46.4)	0.090	0.581
PV, mL	3948.7 ± 531.9 (2830.0–4989.0)	3174.3 ± 359.4 (2563.0–3701.0)	0.000	1.619
PV, mL·kg^−1^	44.32 ± 6.12 (31.3–54.6)	43.24 ± 5.24 (34.6–56.2)	0.582	0.186
PV, mL·FFMkg^−1^	49.94 ± 6.53 (35.7–61.7)	57.23 ± 4.79 (46.9–66.8)	0.001	–1.219
BV, mL	7270.0 ± 716.8 (5788.0–8456.0)	5388.1 ± 470.8 (4620.0–6019.0)	0.000	2.935
BV, mL·kg^−1^	81.62 ± 8.44 (61.70–96.20)	73.51 ± 8.34 (59.80–91.90)	0.006	0.964
BV, mL·FFMg^−1^	91.92 ± 8.39 (72.9–104.6)	97.35 ± 7.57 (84.6–109.4)	0.052	–0.670

Note: [Hb]—haemoglobin concentration; FFM—fat-free mass; Hct—haematocrit; Hb-mass—haemoglobin mass; RBCV—red blood cell volume; PV—plasma volume; BV—blood volume. The ranges of the values are presented in parentheses.

**Table 4:** Correlations between anthropometric and hematological variables in male and female junior rowers.

		Hb-Mass, g	PV, mL	BV, mL	[Hb], g·L^−1^
Males	BM, kg	0.186	0.108	0.152	0.021
	FFM, kg	0.530 **	0.230	0.376	0.115
Females	BM, kg	0.513	0.604 *	0.629 *	–0.388
	FFM, kg	0.492	0.741 **	0.728 **	–0.561 *

Note: Hb-mass—haemoglobin mass; PV—plasma volume; BV—blood volume; [Hb]—haemoglobin concentration; BM—body mass; FFM—fat-free mass; * *p* < 0.05; ** *p* < 0.001.

**Table 5:** Correlations between maximal power values during the graded rowing exercise test and hematological parameters in elite male and female junior rowers.

Males	Hb-Mass, g	Hb-Mass, g·kg^−1^	Hb-Mass, g·FFMkg^−1^	PV, mL	PV, mL·kg^−1^	PV, mL·FFMkg^−1^	BV, mL	BV, mL·kg^−1^	BV, mL·FFMkg^−1^	Hct, %	[Hb], g·L^−1^
Peak power, W	0.418 *	0.263	0.237	0.160	0.071	0.031	0.279	0.148	0.109	0.091	0.099
Peak power, W·kg^−1^	0.216	0.489 *	0.358	0.062	0.248	0.119	0.128	0.365	0.219	0.053	0.062
Peak power, W·FFMkg^−1^	0.059	0.222	0.262	0.001	0.104	0.102	0.022	0.156	0.171	0.013	0.025
Females											
Peak power, W	0.079	0.259	–0.007	0.256	0.484	0.308	0.222	0.423	0.191	–0.303	–0.307
Peak power, W·kg^−1^	–0.338	0.826 **	0.514	–0.362	0.715 **	0.416	–0.388	0.810 **	0.516	0.186	0.192
Peak power, W·FFMkg^−1^	–0.391	0.689 **	0.663 **	–0.482	0.510	0.489	–0.497	0.621 *	0.638 *	0.281	0.287

Note: Hb-mass—haemoglobin mass; PV—plasma volume; BV—blood volume; Hct—haematocrit; [Hb]—haemoglobin concentration; FFM—fat-free mass. * *p* < 0.05; ** *p* < 0.001.

## Data Availability

The data presented in this study were collected specifically for this research and are not publicly available due to ethical and privacy considerations involving minor participants. Anonymized data are available from the corresponding author upon reasonable request.

## Abbreviations

The following abbreviations are used in this manuscript:

[Hb]	Haemoglobin concentration
[La5′]	Blood Lactate Concentration 5 min after Graded Rowing Exercise Test
BV	Blood Volume
FFM	Fat-Free Mass
GRXT	Graded Rowing Exercise Test
Hct	Hematocrit
Hb-mass	Haemoglobin Mass
HR	Heart Rate
RBCV	Red Blood Cell Volume
PV	Plasma Volume
V̇O_2_max	Maximum Oxygen Uptake
